# A Study on Machine Learning Models in Detecting Cognitive Impairments in Alzheimer’s Patients Using Cerebrospinal Fluid Biomarkers

**DOI:** 10.1177/15333175241308645

**Published:** 2024-12-10

**Authors:** Vivek K. Tiwari, Premananda Indic, Shawana Tabassum

**Affiliations:** 1 Department of Electrical & Computer Engineering, 12347 University of Texas at Tyler, Tyler, TX, USA

**Keywords:** machine learning models, Alzheimer’s disease, cerebrospinal fluid, amyloid beta 1-42, P-tau, T-tau, cognitive dementia ratings, Mini-mental state score

## Abstract

Several research studies have demonstrated the potential use of cerebrospinal fluid biomarkers such as amyloid beta 1-42, T-tau, and P-tau, in early diagnosis of Alzheimer’s disease stages. The levels of these biomarkers in conjunction with the dementia rating scores are used to empirically differentiate the dementia patients from normal controls. In this work, we evaluated the performance of standard machine learning classifiers using cerebrospinal fluid biomarker levels as the features to differentiate dementia patients from normal controls. We employed various types of machine learning models, that includes Discriminant, Logistic Regression, Tree, K-Nearest Neighbor, Support Vector Machine, and Naïve Bayes classifiers. The results demonstrate that these models can distinguish cognitively impaired subjects from normal controls with an accuracy ranging from 64% to 69% and an area under the curve of the receiver operating characteristics between 0.64 and 0.73. In addition, we found that the levels of 2 biomarkers, amyloid beta 1-42 and T-tau, provide a modest improvement in accuracy when distinguishing dementia patients from healthy controls.

## Introduction

Alzheimer’s disease (AD) is 1 of the most prevalent neurodegenerative diseases characterized by progressive cognitive decline. This cognitive deterioration also poses a significant burden on the caregivers. Despite Alzheimer’s disease affecting over 55 million people globally,^1^ there remains no reliable and straightforward method for diagnosing the condition. The mild impairment of episodic memory is typically 1 of the first indications of patients with early-stage AD. Although these people may meet the requirements for mild cognitive impairment (MCI) diagnosis, because of their daily life activities are unaffected and their global cognitive functioning is intact, they are not yet considered demented^2,3^ and are not detected in early stages.

Recent research on cerebrospinal fluid (CSF) biomarkers has revealed the potential to detect AD at its early stages.^4,5^ Initial research revealed that T-tau and amyloid beta 1-42 (Aβ_1-42_) together had a good predictive potential to detect early-stage AD in MCI cases.^4^ According to recent studies with prolonged periods of clinical follow-up, the combination of all 3 CSF biomarkers (T-tau, P-tau, and Aβ_1-42_) may have a predictive value as high as 95% to distinguish MCI cases with progression toward AD from stable MCI cases and MCI cases with other types of underlying pathology.^6^ Several large multi-center studies have confirmed that these CSF biomarkers have a strong predictive value for detecting early-stage AD.^2,7,8^

Two types of rating instruments are used to identify dementia subjects. The clinical dementia rating (CDR), first published in 1982,^9^ is considered as the gold standard global rating scale for staging dementia patients. The CDR classifications are zero (no dementia), 0.5 (questionable dementia), 1 (mild dementia), 2 (moderate dementia), and 3 (severe dementia). While the CDR is highly valid and reliable, it depends on a comprehensive structured interview with both the patient and the physician.^10,11^ In addition, the process becomes complicated if a trustworthy and knowledgeable caregiver is unavailable, limiting its utility in everyday practice.^12^ In contrast, simple and shorter assessment techniques such as the Mini-Mental State Examination (MMSE) scores are more appropriate for primary and secondary care levels.^13^ The MMSE score, ranging from zero to 30, is calculated based on a series of questions designed to assess the patient’s cognitive skills. A score of 26 or higher represents normal cognition, scores between 20 and 26 indicate mild dementia, scores between 10 and 20 indicate moderate dementia, and scores less than 9 indicate severe dementia. Numerous studies have demonstrated the reliability and validity of the MMSE score.^14^

Previous research has primarily concentrated on machine learning (ML)-based classification of dementia stages by integrating CSF biomarkers with various other parameters, rather than relying on CSF biomarkers alone.^15^ These studies have explored the synergistic potential of combining CSF biomarkers such as amyloid-beta and tau proteins with clinical assessments, cognitive scores, genetic factors, and imaging data. The primary imaging modalities include Magnetic Resonance Imaging (MRI) and Positron Emission Tomography (PET). For instance, a research project, Alzheimer’s Biomarkers in Daily Practice (ABIDE), utilized the Amsterdam Dementia Cohorts—a longitudinal cohort from a tertiary referral center—comprising 525 individuals.^16^ Each patient’s initial appointment occurred at a clinic between September 1, 1997, and August 31, 2014. The study employed Cox regression (or proportional hazards regression) analysis to develop prognostic models for analyzing the progression of mild cognitive Alzheimer’s phases. The models incorporated MRI biomarkers (hippocampal volume and normalized whole-brain volume), CSF biomarkers (Aβ_1-42_, tau), patient characteristics such as age and gender, and MMSE scores as inputs.

In another work, a support vector machine (SVM) with multi-task learning was applied to predict the 2-year conversion from MCI to AD using baseline MRI and CSF measurements.^17^ The model achieved 73.9% accuracy, 68.6% sensitivity, and 73.6% specificity. Another group of researchers trained a multi-modal Gated Recurrent Unit model to predict the conversion from MCI to AD using longitudinal cognitive performance and CSF biomarkers data, along with cross-sectional neuroimaging and demographic data.^18^ Although these studies demonstrate good accuracy of ML models, their performance relies on imaging data, which are expensive to acquire and require specialized expertise to interpret. Moreover, these imaging modalities often fail to distinguish Alzheimer’s from other neurodegenerative disorders.^19^ In contrast, CSF biomarkers have become increasingly attractive for clinical use. Several research studies demonstrate the potential of Aβ_1-42_ and tau biomarkers in detecting AD pathology in its early stages.^20^

In this work, we studied the performance of ML models for the classification of dementia patients from normal controls based on the CSF biomarkers alone. Various machine learning models such as Discriminant, Logistic Regression, Tree, Support Vector Machine (SVM) and Naïve Bayes classifiers were studied. With 3 CSF biomarkers (Aβ_1-42_, T-tau and P-tau), medium gaussian SVM provided the highest accuracy of 67% with area under the receiver operating characteristic (AUROC) of 0.72, true positive rate (TPR) of 0.78 and false positive rate (FPR) of 0.43. With 2 CSF biomarker levels (Aβ_1-42_ and T-tau), the K-Nearest Neighbor (KNN) classifier model provided the highest accuracy of 69% with AUROC of 0.73, TPR of 0.76 and FPR of 0.37.

## Methods

### Description of Data

We analyzed data from electronic health records of AD patients collected from the National Alzheimer’s Coordinating Center (NACC) database.^21^ CSF biomarker levels from subjects with both CDR and MMSE scores were considered for further analysis, excluding any subjects with missing or erroneous values. CSF biomarker levels were obtained from multiple subjects over several years, and repeated measurements were excluded, resulting in a total of 711 subjects with unique identification numbers. Among the 711 subjects, 356 were categorized as normal controls (NC), 219 as mild, 109 as moderate, and 27 as severe according to MMSE scores. For the same set of subjects, CDR scores categorized 216 as NC, 178 as mild, 29 as moderate, 20 as severe, and 268 as questionable. To make a conservative classification of groups, we included a subject in the dementia group only if it met the criteria for both CDR and MMSE scores, resulting in 210 subjects in the dementia group. To balance the dataset, which is necessary for efficient training and validation of machine learning models, we randomly selected 210 subjects from the NC group who met the criteria for NC using both CDR and MMSE scores.

### Machine Learning Classification Models

We used classification models in MATLAB (MathWorks, R2023a) to train and validate the balanced data set in differentiating dementia subjects from normal control (NC) as a binary classification problem. Since the data set is small, we used 5-fold cross validation to train different types of machine learning models that belong to discriminant, logistic regression, tree, support vector machines and naïve bayes. Models were compared using the performance metrics such as accuracy, area under the receiver operating characteristics (AUROC), true positive rate (TPR) and false positive rate (FPR).

## Results and Discussion

We first studied whether the biomarker levels are statistically different between the dementia and NC groups by considering the balanced data set. Then we studied the correlation of these biomarker levels with the CDR and MMSE scores. Finally, we trained and validated different machine learning models on the data set. By categorizing subjects into dementia and NC groups only when they meet the criteria for both CDR and MMSE scores, we can eliminate any variability in classification based on these scores. Such approach ensures that the performance of the machine learning models relies only on the biomarker levels. A preliminary analysis of machine learning models, including all subjects and the repeated measurements with CDR and MMSE scores considered separately, was reported in our archived work.^22^

Table 1 shows the mean and standard error (SE) of age, CDR, MMSE and 3 biomarker values of dementia and NC groups. These features are significantly different using a s*t* test comparison. Table 2 provides the correlation (denoted by **r**) of biomarker levels with the CDR as well as MMSE scores and shows a significant weak correlation. As expected, the correlation coefficient values are opposite in sign between CDR and MMSE scores.

**Table 1.** Mean (SE) of Dementia and NC Groups.

Variables	Dementia (n = 210)	NC (n = 210)
Age (years)	70.20 (0.67)	65.95 (0.51)
MMSE	17.91 (0.42)	29.01 (0.05)
CDR	1.32 (0.04)	0.17 (0.01)
Aβ_1-42_ (pg/ml)	227.8 (−10.29)	417.93 (−12.36)
T-tau (pg/ml)	312.26 (36.06)	157.02 (8.48)
P-tau (pg/ml)	44.68 (2.03)	37.39 (1.17)

**Table 2.** Correlation of Biomarker Levels With MMSE and CDR Scores. All Features are Significantly Different Using a Studentst test Statistics With a p-value Less Than 0.001.

Biomarkers	MMSE	CDR
Aβ_1-42_ (pg/ml)	*r* = 0.34	*r* = −0.38
	*P* < 0.001	*P* < 0.0001
T-tau (pg/ml)	*r* = −0.15	*r* = 0.21
	*P* < 0.001	*P* < 0.0001
P-tau (pg/ml)	*r* = −0.12	*r* = 0.11
	*P* < 0.01	*P* < 0.01

Table 3 outlines the performance of machine learning models in classifying NC from dementia patients. Various models using 3 biomarker levels show comparable accuracy, ranging from 64% to 67%. AUROC, TPR and FPR metrics are also comparable. Hence, any of these models can be used to differentiate dementia from NC. When the models were trained exclusively with MCI subjects and NC, while further balancing the dataset, the results remained consistent. This suggests that the model’s performance is influenced by the biomarker levels of MCI subjects that are significantly more prevalent in the dementia group. Adding age, gender, sex, and race as additional features did not significantly alter the model performance metrics.

**Table 3.** Performance of Machine Learning Models for Two Classes (NC Versus Dementia).

	Three Biomarkers NC Vs Dementia				Two Biomarkers NC Vs Dementia			
Model	Accuracy (%)	AUROC	TPR	FPR	Accuracy (%)	AUROC	TPR	FPR
Linear discriminant	66	.71	.70	.39	67	.72	.71	.38
Efficient logistic regression	65	.72	.69	.38	67	.72	.71	.37
Coarse tree	65	.66	.80	.50	63	.64	.78	.49
Medium Gaussian svm	67	.72	.78	.43	67	.73	.70	.36
Medium knn	64	.68	.73	.45	69	.73	.76	.37

Feature importance scores sorted using Analysis of Variance (ANOVA) algorithm showed that Aβ_1-42_ had a score of 12.23 and T-tau had a score of 12.89 whereas P-tau had the lowest value of 3.73 suggesting that Aβ_1-42_ and T-tau biomarker levels are contributing significantly to the performance of the machine learning models in classification. Further confirmation is obtained by checking the feature importance scores sorted using Kruskal Wallis algorithm in which Aβ_1-42_ had a score of 14.88, T-tau had a score of 14.02 and P-tau had a score of 6.15.

**Figure 1.**
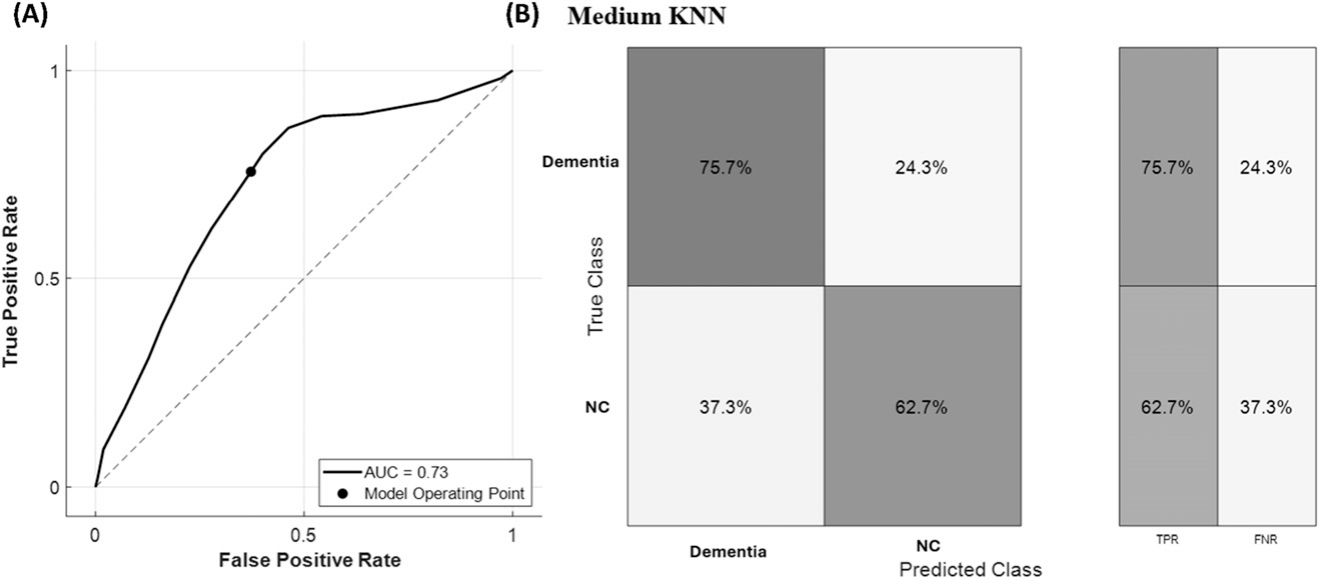
Performance metrices of the best performance model using two biomarker levels. (A) Reciever operating charateristics and (B) Confusion matrix along with TPR and FNR values.

Based on the findings from the feature important scores, we trained and validated the models with 2 biomarker levels, Aβ_1-42_ and T-tau, as shown in Table 3. The performance metrics of the models are comparable with the models with 3 biomarker levels confirming the earlier observations.^4^ Figure 1(A) shows the ROC curve for differentiating NC from dementia based on 2 biomarkers, with the operating point (shown as the dot) set at a TPR of 0.76 and FPR of 0.37. The model achieves an AUROC of 0.73. Figure 1(B) shows the confusion matrix, highlighting the TPR for detecting dementia and NC groups, along with their FNR. The TPR suggests that the dementia patients can be detected with a sensitivity of 76% and FPR suggests a specificity of 63%.

In summary, CSF biomarkers alone offer potential in the early detection of AD. The results are encouraging from a future standpoint because they show good accuracy with fewer input features, in this case 2 biomarkers. The model’s accuracy could be improved by including more patient data and real-time input features. There is evidence in the literature that a strong correlation exists between blood biomarkers and CSF biomarkers.^23^ Recent research indicates that a blood test measuring tau and the Aβ_42_/Aβ_40_ ratio demonstrates high diagnostic accuracy (range, 88%-92%) for identifying Alzheimer’s disease in both primary and secondary care settings.^24^ Hence, future research could also involve developing a point-of-care blood sampling device interfaced with machine-learning classification models for early diagnosis and prognosis of Alzheimer’s disease patients.

## Data Availability

The datasets generated and/or analyzed during this study may be available from the corresponding author upon reasonable request.*
